# Proteomic profiling of prostate cancer reveals molecular signatures under antiandrogen treatment

**DOI:** 10.1186/s12014-024-09490-9

**Published:** 2024-06-26

**Authors:** Yurun Huang, Guanglin Yang, Xinpeng Yao, Yue Fang, Qiliang Lin, Menghan Zhou, Yiping Yang, Qinggui Meng, Qingyun Zhang, Shan Wang

**Affiliations:** 1 Department of Research, Guangxi Medical University Cancer Hospital, Nanning, Guangxi China; 2 Department of Urology, Guangxi Medical University Cancer Hospital, Nanning, Guangxi China; 3 The First Clinical Medical College, Guangxi Medical University, Nanning, Guangxi China

**Keywords:** Prostate Cancer, Antiandrogen, Diagnostic biomarkers, Proteomics, Deubiquitinase, E3 ubiquitin ligase

## Abstract

**Background:**

Tumorigenesis and progression of prostate cancer (PCa) are indispensably dependent on androgen receptor (AR). Antiandrogen treatment is the principal preference for patients with advanced PCa. However, the molecular characteristics of PCa with antiandrogen intervention have not yet been fully uncovered.

**Methods:**

We first performed proteome analysis with 32 PCa tumor samples and 10 adjacent tissues using data-independent acquisition (DIA)- parallel accumulation serial fragmentation (PASEF) proteomics. Then label-free quantification (LFQ) mass spectrometry was employed to analyze protein profiles in LNCaP and PC3 cells.

**Results:**

M-type creatine kinase CKM and cartilage oligomeric matrix protein COMP were demonstrated to have the potential to be diagnostic biomarkers for PCa at both mRNA and protein levels. Several E3 ubiquitin ligases and deubiquitinating enzymes (DUBs) were significantly altered in PCa and PCa cells under enzalutamide treatment, and these proteins might reprogram proteostasis at protein levels in PCa. Finally, we discovered 127 significantly varied proteins in PCa samples with antiandrogen therapy and further uncovered 4 proteins in LNCaP cells upon enzalutamide treatment.

**Conclusions:**

Our research reveals new potential diagnostic biomarkers for prostate cancer and might help resensitize resistance to antiandrogen therapy.

**Supplementary Information:**

The online version contains supplementary material available at 10.1186/s12014-024-09490-9.

## Introduction

Prostate cancer (PCa) is the second leading cause of male cancer-related deaths in the USA [1] and the fifth most common cause of cancer deaths worldwide [2]. Although there are shortfalls, prostate-specific antigen (PSA) testing substantially contributes to early PCa detection and a declining mortality rate in developed countries, which should be further conducted in China [3]. As the critical molecules for cell viability and function, aberrant proteins could be the ultimate cause of cancer or key targets to treat cancer. Deep mining of the cancer proteome could help to understand the mechanism of tumorigenesis and discover novel biomarkers or potential therapeutic targets. In PCa, proteomics was applied to discover biomarkers [4–6], key proteins in PCa progression [7, 8], or protein interaction networks [9, 10]. In recent years, the application of 4-dimensional proteomics has provided a promising tool to identify more proteins with lower abundance due to its high sensitivity, compared with traditional 3D proteomics [11].

Precise regulation of protein turnover is pivotal for cellular health. E3 ubiquitin ligases and DUBs are key regulators for protein degradation. There are more than 600 E3 ligases (E3 ubiquitin ligome) and 100 DUBs (DUBome) in human cells [12, 13]. Exploring the expression changes of E3 ligases and DUBs could be conducive to analyzing proteostasis and tumorigenesis in PCa.

Enzalutamide and bicalutamide, two important antiandrogen drugs, target the core protein AR in PCa. Compared with first-generation antiandrogen bicalutamide, second-generation antiandrogen enzalutamide could benefit patients with non-metastatic castration-resistant prostate cancer (CRPC) and metastatic prostate cancer [14, 15]. However, patients could eventually develop enzalutamide resistance [16]. Currently, several mechanisms related to enzalutamide resistance have been reported, such as AR variants [17], transcriptional profiling alteration [18], immune evasion [19], and lncRNA [20]. Nonetheless, advanced proteomics analysis of PCa clinical samples and cells upon antiandrogen treatment could furnish comprehensive information about protein expression and protein interaction networks for investigating the molecular mechanism of enzalutamide resistance.

In this study, we conducted a proteomics investigation of PCa, evaluated the E3 ubiquitin ligome and DUBome in PCa clinical samples, and discovered two potential diagnostic biomarkers of PCa. Furthermore, the proteome in PCa patients under bicalutamide treatment was scrutinized. Finally, by comparing the proteomes of PCa samples and cells, we figured out several proteins that have the potential to be biomarkers of antiandrogen treatment, which could help to develop novel compounds to sensitize enzalutamide resistance in advanced PCa.

## Materials and methods

### Experimental design

This study was designed to discover the proteomic landscape of PCa and the change of proteome in PCa upon antiandrogen intervention. In this cohort, 32 local tumor samples (tumor group) and 10 normal tissues adjacent to tumor (control group) were collected from the Guangxi Medical University Cancer Hospital. The patients with PCa were histopathologically diagnosed. The clinical samples were snap-frozen in liquid nitrogen immediately after collection. Detailed information on clinical samples is presented in Additional file 7: Table S1.

### Protein extraction and trypsin digestion

The samples were weighed into a liquid nitrogen pre-cooled mortar and ground to power with liquid nitrogen. Each sample was added with 4x volume of lysis buffer (1% SDS, 1% protease inhibitor cocktail) of the powder and lysed by ultrasonication on ice using a high-intensity ultrasonic processor (Scientz). The samples were centrifuged at 12,000 g for 10 min at 4 °C, and the supernatant was transferred to a new centrifugation tube. The protein concentration was determined by using the bicinchoninic acid (BCA) kit.

An equal amount of each sample protein was taken for enzyme digestion, and the volume was adjusted to consistency with lysis buffer. One volume of pre-cooled acetone was added to sample, vortexing and mixing, then 4x volume of pre-cooled acetone was appended, and samples were kept at -20 °C for 2 h for protein precipitation. Centrifugation was performed at 4,500 g for 5 min, discarding the supernatant, and the precipitate was washed with pre-cooled acetone for 2–3 times. After drying the precipitate, add a final concentration of 200 mM tetraethylammonium bromide (TEAB) and ultrasonically disperse. Trypsin was added at a 1:50 trypsin-to-protein mass ratio for the first digestion overnight. Then sample was reduced with 5 mM dithiothreitol (DTT) for 30 min at 56 °C and alkylated with 11 mM iodoacetamide (IAA) for 15 min at room temperature in darkness. Finally, the peptides were desalted by the Strata X SPE column.

### LC-MS/MS analysis

The tryptic peptides were dissolved in solvent A and directly loaded onto a homemade reversed-phase analytical column (25 cm length, 100 μm i.d.). The mobile phase contained solvent A (0.1% formic acid and 2% acetonitrile in water) and solvent B (0.1% formic acid and 90% acetonitrile in water). Peptides were segregated at a constant flow rate of 700 nl/min on an EASY-nLC 1200 UPLC system (ThermoFisher Scientific). The separated peptides were detected in Orbitrap Exploris 480 with a nano-electrospray ion source. Precursors and fragments were determined at the Orbitrap detector. The full MS scan resolution was set to 60,000 at a scan range of 350–1400 m/z. The MS/MS scan was fixed initial mass at 120.0 m/z with a resolution of 15,000. Data acquisition mode was accommodated to data-independent acquisition (DIA) program. The HCD fragmentation was conducted with a normalized collision energy (NCE) of 27%. Automatic gain control (AGC) was set to 1E6, with a maximum injection time of 22 ms.

### Database search

The DIA-NN search engine (v.1.8) was employed to process the DIA data. Tandem mass spectra were searched using Homo_sapiens_9606_SP_20230103.fasta (20,389 entries) concatenated with the reverse decoy database. As a cleavage enzyme, trypsin/P was designated as permitting up to one missing cleavage. The fixed modifications were carbamidomethyl on Cys and excision on N-term Met. The FDR was changed to < 1%.

### Bioinformatics methods

The ratio of the average protein abundances in each group was utilized to calculate the protein fold change (FC) between the tumor group and the control group. The *P* values were computed using the Student’s t-test. Proteins with fold change (FC) > 1.5 and *P* values < 0.05 were defined as differential expression proteins (DEPs). Gene ontology (GO) annotation is the process of extracting the GO ID of each identified protein using eggnog-mapper software (v2.1.6) based on the EggNOG database and then annotating the proteins according to cellular components, molecular functions, and biological processes [21]. The Kyoto Encyclopedia of Genes and Genomes (KEGG) database was referred to annotate protein enrichment pathways using Diamond (v2.0.11.149), and proteins were identified through BLAST comparison (Blastp, E-value ≤ 1e-4). Based on one-way hierarchical clustering (Euclidean distance, average linkage clustering) in Genesis, heap maps were visualized by using the “Heatmap” function from the “ComplexHeatmap” R-package. Volcano plots were created using the R package ggplot2 (version 3.3.5). Protein-protein interactions (PPI) of DEPs were searched using the STRING database. PPI was visualized by the plugin yFiles Layout Algorithms (v1.1.3) in Cytoscape software (v3.10.0) [22].

### Cell culture and enzalutamide treatment

AR-positive LNCaP cells and AR-negative PC3 cells were maintained in RPMI medium with 10% FBS (v/v), 100 µg/ml streptomycin and 100 U/ml penicillin. Cells were cultured at 37 °C in a humidified incubator with 5% CO_2_. For the antiandrogen intervention, cells were treated with 10 µM enzalutamide for 24 h and were snap-frozen in liquid nitrogen for proteomic analysis. Also, cells were treated with 10 µM bicalutamide for 24 h for immunoblots detection.

### Label-free quantification (LFQ) mass spectrometry

For cell samples, LFQ MS was employed to scrutinize change in the proteome. Three replicates of each treatment were used. Protein extraction and trypsinization of cell samples followed the same protocol for clinical samples.

Peptides were separated at a constant flow rate of 450 nL/min using a NanoElute ultra-high performance liquid chromatography system after being solubilized with mobile phase A (0.1% formic acid and 2% acetonitrile in water). Mobile phase B contained 0.1% formic acid and 100% acetonitrile. Peptides separated by the UHPLC system were injected into a Capillary ion source for ionization and then into a timsTOF Pro mass spectrometer. The ion source voltage was set at 1.75 kV, and the peptide parent ions and their secondary fragments were detected and analyzed by high-resolution TOF. The scanning range of the secondary mass spectra was set to 100–1700. The data acquisition mode used was parallel accumulation serial fragmentation (PASEF) mode. One primary mass spectrum was acquired, followed by 10 secondary spectra in PASEF mode with parent ion charges in the range of 0–5, and the dynamic exclusion time of the tandem mass spectrometry scans was set to 30 s to avoid repetitive scans of the parent ions.

Secondary mass spectrometry data for this experiment were retrieved using Maxquant (v1.6.15.0). The searching database was Homo_sapiens_9606_SP_20201214.fasta (20,395 sequences). The decoy database was added to calculate the false-positive rate (FDR) caused by random matching, and the common contaminant proteins were added to the database to eliminate the influence of contaminant proteins. Carbamidomethyl (C) was set as a fixed modification, and the variable modifications were oxidation of methionine and acetylation of the N-terminus of the protein. The FDR for protein identification and peptide-spectrum match (PSM) identification was set to 1%.

The bioinformatic analysis of MS data from cell samples was completed using the same programs for clinical samples.

### Gene expression data, survival data and single-cell RNAseq data

Gene expression data and survival data of selected proteins in the Cancer Genome Atlas (TCGA) prostate cohort and gene expression data of proteins in GTEx (Genotype-Tissue Expression) normal prostate tissue were obtained from the UCSC Xena platform [23]. Single-cell RNAseq data GSE120716 of normal prostate was acquired from NCBI [24].

### Statistical analysis

Gene expression data, survival data, and ROC curves from proteomics, TCGA, or GTEx were visualized with GraphPad Prism. Unpaired Student’s t-test was used to investigate two sets of data. Asterisks denote significant differences (**** *P* < 0.0001; *** *P* < 0.001; ** *P* < 0.01; * *P* < 0.05; ns, not significant).

## Results

### Overview of proteome in clinical samples of prostate cancer

In this study, DIA-based proteomics was employed to scrutinize the proteome alterations in PCa patients. Totally, with 1% FDR at protein levels, 77,090 peptides and 8,771 proteins were identified, and 8,551 (97.5%) comparable proteins in both PCa and control samples were determined (Additional file 1: Figure S1A, Additional file 7: Table S2). To investigate the sample quality, the distributions and differentiation of protein intensity in each sample were presented (Additional file 1: Figure S1B). The similar violin area of all samples and mean values of Log10 intensity indicate good sample quality. All identified proteins were annotated in Gene Ontology (GO) (95.3%), COG/KOG (79.6%), Reactome (66.5%), and KEGG pathway (47.9%) (Additional file 1: Figure S1C).

### Functional classification and enrichment analyses and protein-protein interaction networks in csDEPs

**Fig. 1:**
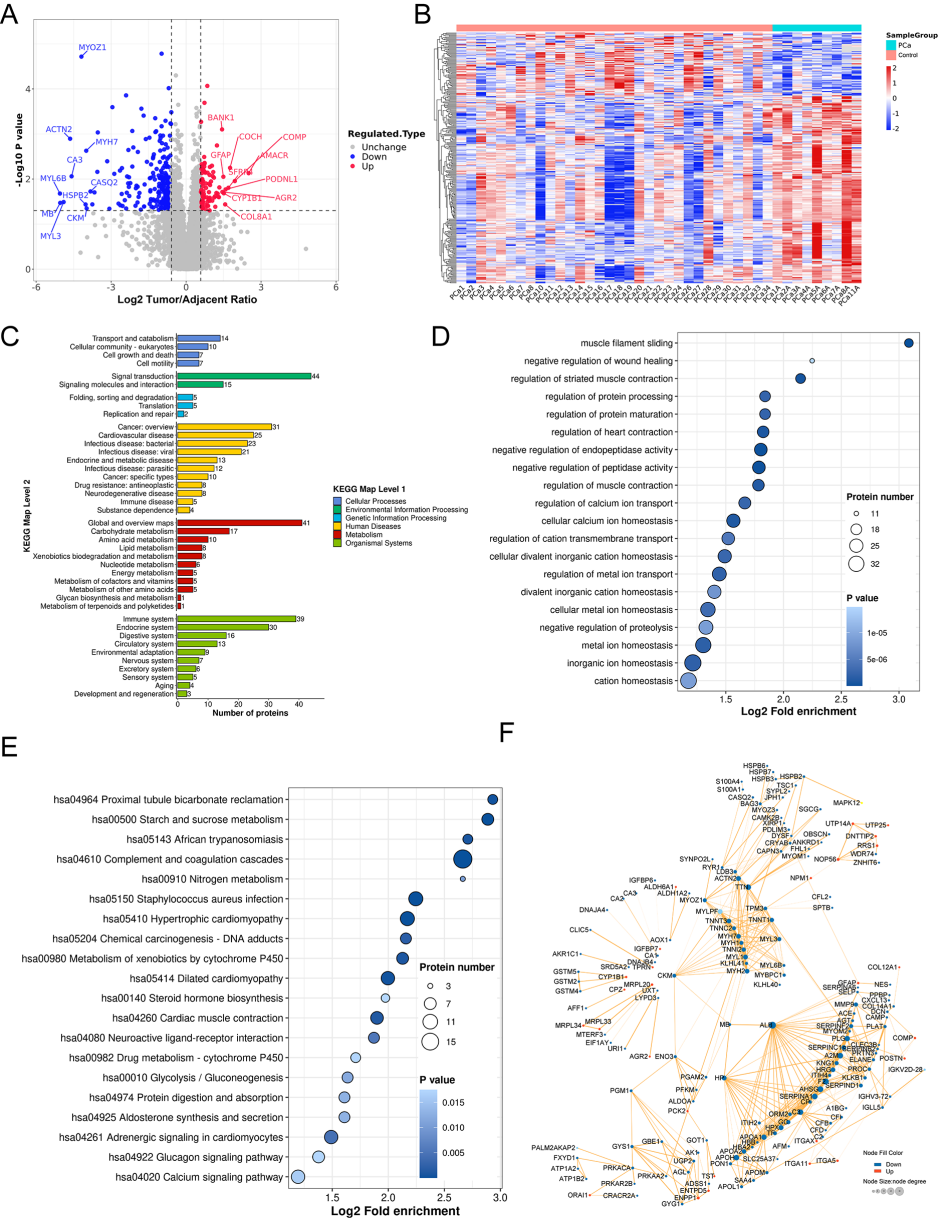
Bioinformatical analysis of csDEPs between the PCa samples and the adjacent tissues. **A** Volcano plot of all identified proteins. Up-regulated proteins were set as red dots and down-regulated proteins were blue dots. The most changed proteins were labeled. **B** Heatmap of csDEPs among all PCa samples and adjacent tissues. **C** Functional classification of KEGG pathway for csDEPs. **D** Dot plot of gene ontology (GO) enrichment of csDEPs in biological process (BP). **E** Dot plot of pathway enrichment of csDEPs in KEGG pathway. **F** Protein-protein interaction (PPI) network of csDEPs visualized by Cytoscape. Blue dots for down-regulated proteins and red dots for up-regulated proteins. Node size was set from node degree. csDEPs, differential expression proteins in clinical samples

To evaluate significant changes in the proteome in PCa, we identified 89 up-regulated and 272 down-regulated proteins in PCa group with fold-change > 1.5 and *P* value < 0.05 (Fig. 1A, Table S3). The hierarchical clustering of clinical sample DEPs (csDEPs) demonstrated a relatively significant difference between PCa group and control group (Fig. 1B). Also, varied expression levels of protein in each group indicated the individual differences in proteome. Then, the functional classification and enrichment of csDEPs were conducted. Metabolism, diseases and signal transduction-related proteins in KEGG maps were major classifications in csDEPs (Fig. 1C). In GO enrichment analysis, regulation of enzymatic activity, ion homeostasis and regulation of ion transport are enriched in biological process (BP) (Fig. 1D). Furthermore, pathway analysis revealed that the significant enrichment pathways of csDEPs in KEGG included complement and coagulation cascades, metabolism of cytochrome P450, starch and sucrose metabolism and so on (Fig. 1E). The significant changes of these pathways and corresponding proteins in PCa hint their important roles in PCa tumorigenesis and progression.

Next, we generated protein-protein interaction network of csDEPs with integrated data from STRING database and visualized in Cytoscape (Fig. 1F). To find biological networks in csDEPs, Markov cluster algorithm (MCL) was employed. We identified two clusters (muscle protein and myofibril assembly, microfilament motor activity and actin binding) and several proteins associated with mitochondrial translation (MRPL20, MRPL33, MRPL34), RNA processing (RRS1, NPM1, DNTTIP2), biological oxidations (CFL2, PGM1, GSTM2), ECM organization (COL14A1), (Additional file 2: Figure S2A). This finding aligns with prior research demonstrating these proteins are differentially expressed in tumor tissues [25]. Interestingly, two clustering networks (Complement and coagulation cascades, starch and sucrose metabolism) were recognized in csDEGs (Additional file 2: Figure S2A). The complement and coagulation cascades-associated proteins have the most interacting partners in csDEPs, and all the proteins in this pathway were downregulated in PCa (Fig. 1F), implying potential targets to reprogram the immune cold status of PCa.

Finally, we checked the most significantly altered csDEPs and found a down-regulated creatine kinase CKM and an up-regulated extracellular matrix (ECM) protein COMP at both protein and mRNA levels (Additional file 2: Figure S2B, S2E). Survival data demonstrated that high expression level of *CKM* and low expression level of *COMP* improved relapse-free survival (RFS) of PCa in TCGA cohort (Additional file 2: Figure S2F, S2I). ROC analysis suggested that both CKM and COMP have the potential to be diagnostic biomarkers at mRNA and protein levels for high-risk primary PCa (Additional file 2: Figure S2G, S2H, S2J, S2K).

### E3 ligome and DUBome in PCa samples

**Fig. 2:**
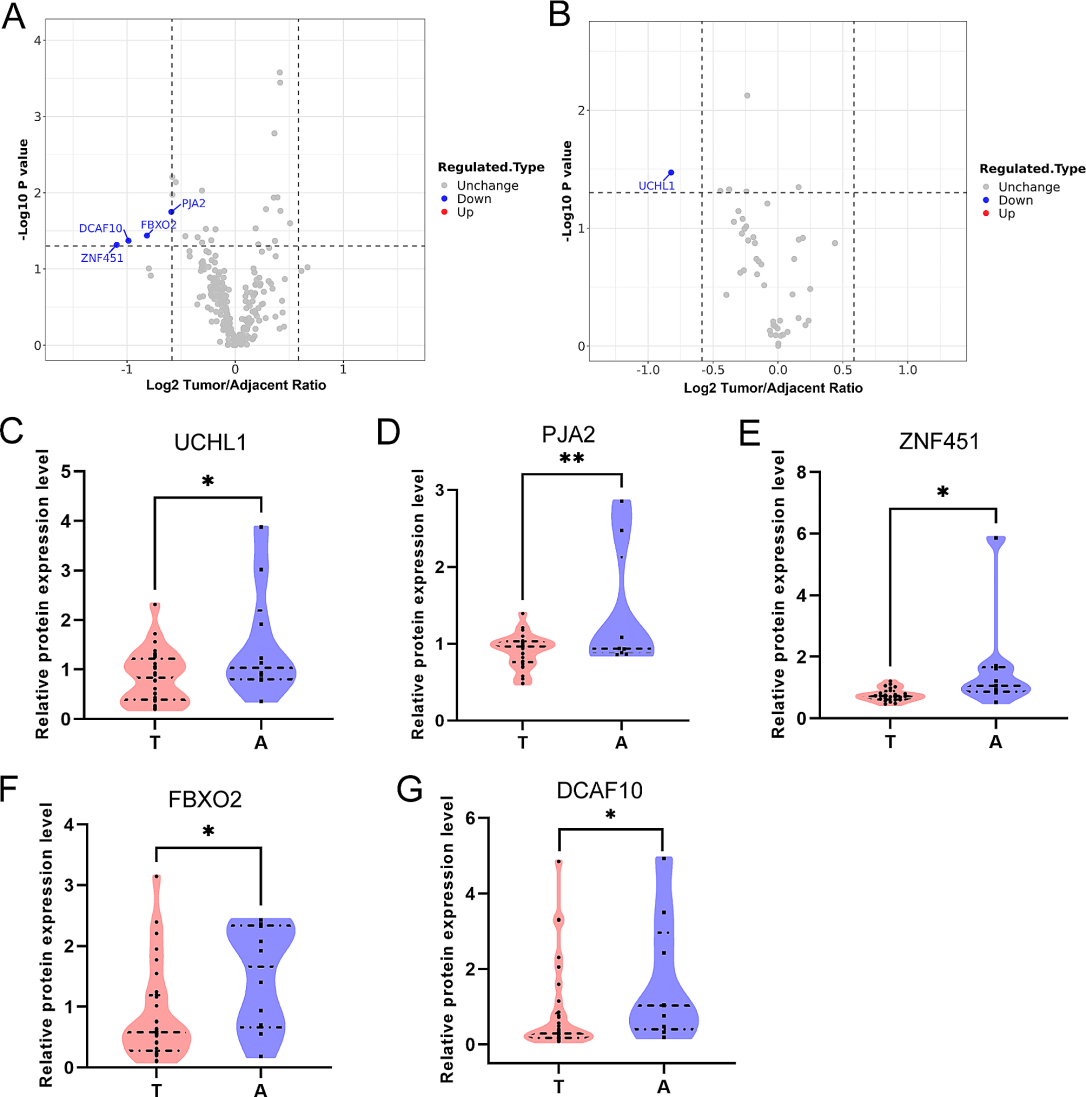
E3 ligome and DUBome in PCa samples. **A** Volcano plot of E3 ligome in PCa samples, Tumor/Adjacent. Down-regulated proteins were set as blue dots. **B** Volcano plot of DUBome in PCa samples, Tumor/Adjacent. Blue dot indicated down-regulated protein. **C-G** Protein expressions of UCHL1, PJA2, ZNF451, FBXO2, and DCAF10 in PCa tumors and adjacent tissues

E3 ubiquitin ligases and DUBs play important roles in the regulation of protein turnover and the maintenance of proteostasis [26]. We selected E3 ligome (Fig. 2A) and DUBome (Fig. 2B, Additional file 7: Table S4) in PCa proteome and found five significantly downregulated proteins, including E3 ligases PJA2, ZNF451, FBXO2 and DCAF10, and deubiquitinase UCHL1 (Fig. 2C and G). And we also analyzed the TCGA data and found that the mRNA expression levels of *UCHL1* and *PJA2* in tumor are lower than those in normal tissue (Additional file 3: Figure S3A, S3B), consistent with their protein expressions (Fig. 2C, D). Survival data in PCa showed that low expression of *UCHL1* and high expression of *PJA2* benefit prognosis of patients (Additional file 3: Figure S3C, S3D). ROC analysis indicated mRNA expression of *UCHL1* and *PJA2* as potential diagnostic biomarkers for primary PCa (Additional file 3: Figure S3E, S3F).

### Identification of discovered potential biomarkers

**Fig. 3:**
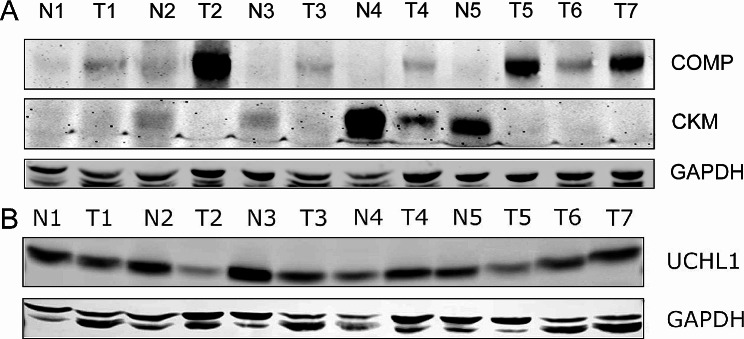
Identification of protein expression in PCa. **A** Immunoblots of COMP, CKM and GAPDH in PCa. **B** Immunoblots of UCHL1 and GAPDH in PCa

To identify the detected potential biomarkers, we performed immunoblots by using specific antibodies. As shown in Fig. 3A, COMP was up-regulated and CKM was down-regulated in PCa tumors, further confirming the potential of COMP and CKM as diagnostic biomarkers for PCa. DUB UCHL1 was down-regulated in PCa, similar to the proteomic data (Fig. 3B). Curiously, the IHC staining of UCHL1 in PCa is quite weak from Human Protein Atlas (HPA) (Additional file 3: Figure S3G). For the E3 ligase PJA2, the IHC staining intensity in normal prostate tissue is concentrated in the moderate range, while in prostate cancer (PCa) it is more commonly weak, negative, or barely strong (Additional file 3: Figure S3H-S3I) (www.proteinatlas.org) [27]. These data provide evidence to support the findings of proteomics in PCa.

### Outline of proteome in PCa cells under enzalutamide treatment

Next, to investigate the change of proteome in PCa cells with an androgen-receptor inhibitor and for the less complexity of cell sample than tumor tissue, we used label-free quantification (LFQ) mass spectrometry to detect protein profiles in androgen receptor (AR)-positive LNCaP cells and AR-negative PC3 cells under enzalutamide treatment (Additional file 4: Figure S4A). The similar distributions and differentiation of protein intensity in each sample (Additional file 4: Figure S4B) and relative standard deviation (RSD) boxplots of samples (Additional file 4: Figure S4C) demonstrated good quality and repeatability. With 1% FDR at protein levels, 95,764 peptides, and 7,943 proteins were identified, and 7,284 (91.7%) comparable proteins in all samples were determined (Additional file 4: Figure S4D, Additional file 7: Table S5). And identified proteins were annotated in COG/KOG (83.0%), Gene Ontology (GO) (72.4%), Domain (56.6%) and KEGG pathway (46.1%) (Additional file 4: Figure S4E).

### Proteomic characteristics of PCa cells after the administration of enzalutamide

**Fig. 4:**
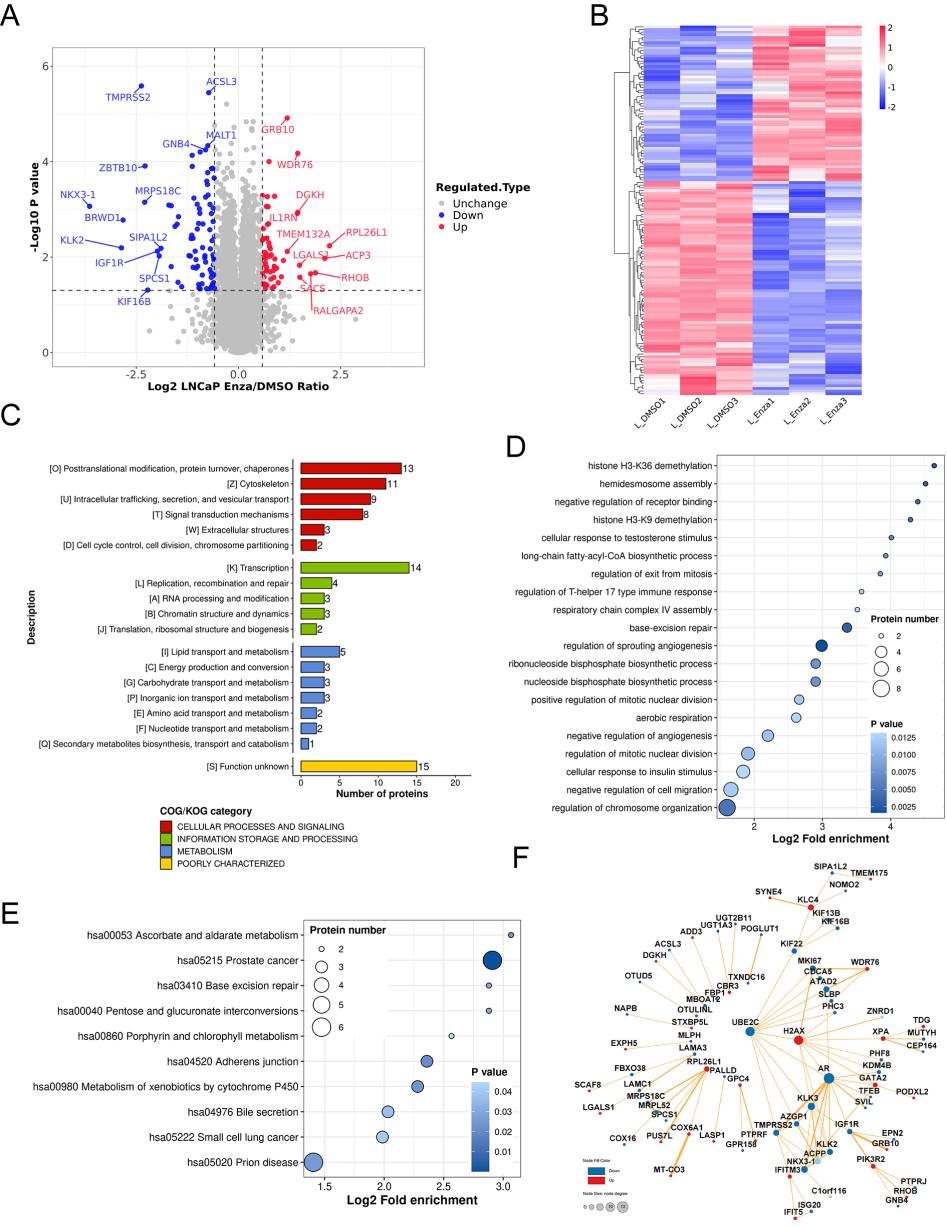
Proteomic features of LNCaP cells after the administration of enzalutamide. **A** Volcano plot of all identified proteins in LNCaP cells. Red dots indicated up-regulated proteins and blue dots for down-regulated proteins. The top changed proteins were labeled. **B** Heatmap of lnDEPs. **C** COG/KOG classification of lnDEPs. **D** Dot plot of gene ontology (GO) enrichment of lnDEPs in biological process (BP). **E** Dot plot of pathway enrichment of lnDEPs in KEGG pathway. **F** Protein-protein interaction (PPI) network of lnDEPs visualized by Cytoscape. Blue dots for down-regulated proteins and red dots for up-regulated proteins. Node size was set from node degree. lnDEPs, differential expression proteins in LNCaP cells

Enzalutamide could favor overall survival in patients with castration-resistant prostate cancer. After 24 h treatment, 81 down-regulated and 59 up-regulated proteins with a fold-change > 1.5 and p value < 0.05 in LNCaP cells and 17 down-regulated and 16 up-regulated proteins in PC3 cells were detected (Fig. 4A, B; Additional file 5: Figure S5A, S5B, and Additional file 7: Table S6). The differential numbers of DEPs in LNCaP cells (lnDEPs) and PC3 cells (pDEPs) could be caused by AR signaling pathway. Some AR downstream genes, such as TMPRSS2, KLK2, and NKX3-1, were significantly decreased in LNCaP cells under enzalutamide treatment. COG/KOG classification of lnDEPs was [O]posttranslational modification, protein turnover, chaperones, [Z]cytoskeleton, [K]transcription and so on, while COG/KOG category of pDEPs was [T]signal transduction mechanisms, [J]translation, ribosomal structure and biogenesis, [C]energy production and conversion, et al. (Fig. 4C and Additional file 5: Figure S5C). In GO biological process, lnDEPs were enriched in histone demethylation, negative regulation of receptor binding, cellular response to testosterone stimulus, base-excision repair, and so forth, and pDEPs were concentrated in telomere maintenance, negative regulation of wound healing, nucleosome assembly, regulation of DNA repair etc. (Fig. 4D and Additional file 5: Figure S5D). Furthermore, KEGG pathway analysis manifested that prostate cancer, adherent junction, and metabolism of xenobiotics by cytochrome P450 were the principal pathways of lnDEPs (Fig. 4E), and RIG-I-like receptor signaling pathway, oxidative phosphorylation, and thermogenesis were main for pDEPs (Additional file 5: Figure S5E).

According to PPI network of lnDEPs, AR has the most interaction partners (Fig. 4F), which further indicates the core role of AR in LNCaP cells. However, due to lack of AR, there should be other effectors in PC3 cells upon enzalutamide treatment. COX7C and NUDFA12 in oxidative phosphorylation pathway and H2AX and RAD51 in regulation of DNA repair could be the effectors of enzalutamide in PC3 cells (Additional file 5: Figure S5F). Interestingly, H2AX was increased in both LNCaP cells and PC3 cells. DNA repair-related proteins H2AX, XPA, and TDG in lnDEPs H2AX and RAD51 in pDEPs indicated that enzalutamide treatment could lead to different DNA damage and repair pathways in LNCaP cells and PC3 cells.

### E3 ligome and DUBome in PCa cells under treatment with enzalutamide

**Fig. 5:**
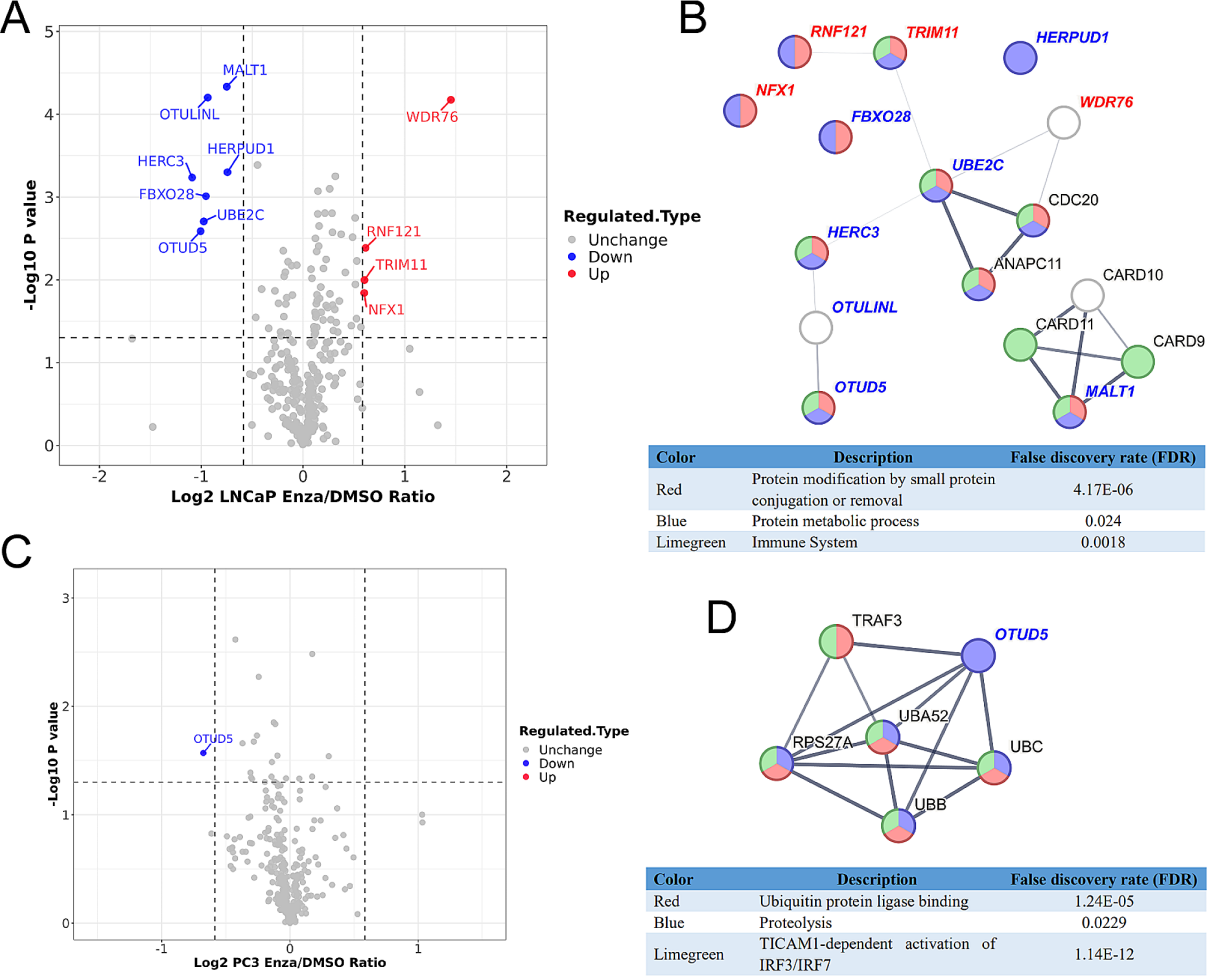
E3 ligome and DUBome in PCa cells treated by enzalutamide. **A** Volcano plot of E3 ligome and DUBome in LNCaP cells. Down-regulated proteins were set as blue dots and up-regulated as red. **B** PPI network evaluated by STRING of significantly changed E3 ligases and DUBs in LNCaP cells. **C** Volcano plot of E3 ligome and DUBome in PC3 cells. Blue dot for down-regulated protein. **D** PPI network generated by STRING for significantly altered DUB OTUD5 in PC3 cells

In lnDEPs (Additional file 7: Table S7), 7 down-regulated and 4 up-regulated E3 ligases and DUBs were detected (Fig. 5A). E3 ligase WDR76 was found to have interaction with MKI67, ATAD2, and SLBP in lnDEPs network (Fig. 4F), and increased WDR76 could be the reason for the reduction of these three proteins upon enzalutamide treatment. We built a PPI network with 11 E3 ligases and DUBs on STRING database and found that most of them participate in protein modification, protein metabolic process, or immune system (Fig. 5B). In pDEPs, OTUD5 was only significantly changed DUB in PC3 cells upon enzalutamide treatment (Fig. 5C). PPI network of OTUD5 from STRING database exhibits its interacting partners (Fig. 5D). OTUD5 in both lnDEPs and pDEPs hints that it could be an effector protein of enzalutamide independent of AR.

### The effect of antiandrogens on proteome of PCa

**Fig. 6:**
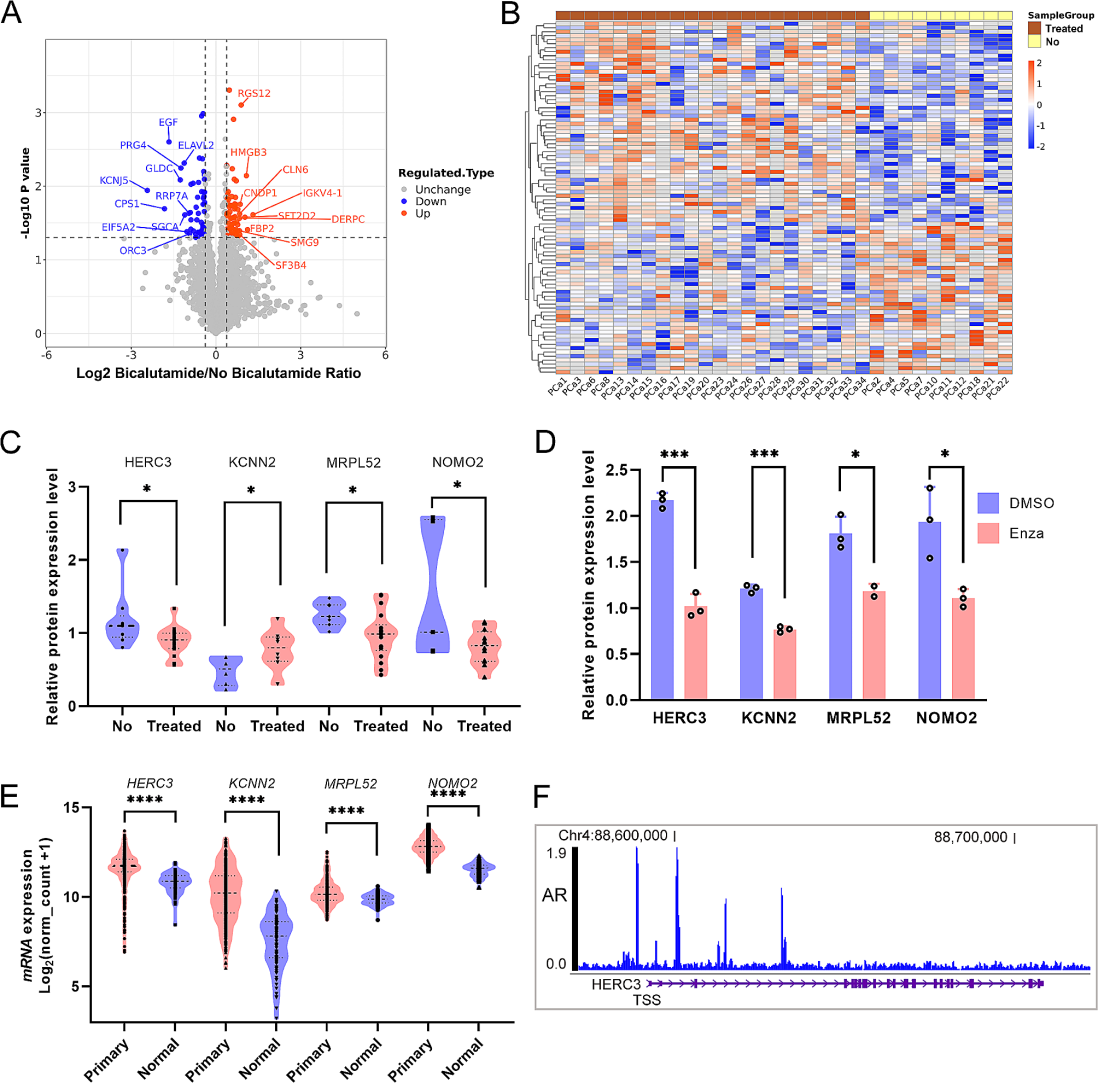
Potential prediction biomarkers for PCa under antiandrogen treatment. **A** Volcano plot of identified proteins in PCa samples with bicalutamide intervention. Red dots for up-regulated proteins and blue dots for down-regulated proteins. **B** Heatmap of differential expression proteins in bicalutamide-treated PCa. **C** Expression levels of HERC3, KCNN2, MRPL52, and NOMO2 proteins in PCa tumors with or without bicalutamide administration. **D** Expression levels of HERC3, KCNN2, MRPL52, and NOMO2 proteins in LNCaP cells treated by enzalutamide. **E** Expression of mRNA levels of *HERC3*, *KCNN2*, *MRPL52*, and *NOMO2* in PCa tumors and adjacent tissues. **F** AR occupancy is increased at the putative promoter region of *HERC3* in the WashU browser for AR ChIP-seq data

**Fig. 7:**
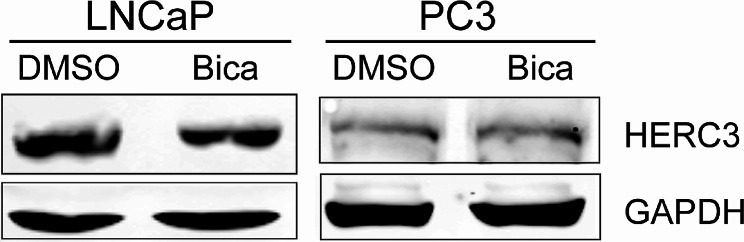
Identification of HERC3 protein expression in LNCaP cells and PC3 cells with bicalutamide treatment

To analyze the effect of antiandrogens on proteome of PCa, we divided PCa samples into two groups, treated group with bicalutamide and non-treated group. We adjusted the proteins with a fold-change > 1.3 and *P* value < 0.05 as significantly differential proteins and found 68 up-regulated and 59 down-regulated proteins upon bicalutamide treatment (Fig. 6A, Additional file 7: Table S8). Hierarchical clustering of the two groups demonstrated a significant difference (Fig. 6B). The expression level of AR protein did not show a significant change (Additional file 6: Figure S6A) in PCa samples under bicalutamide treatment. However, the expression level of AR protein significantly decreased in LNCaP cells upon enzalutamide treatment (Additional file 6: Figure S6B). Then, to extract significantly changed proteins with antiandrogen administration, we compared the DEPs in PCa samples and LNCaP cells and targeted four proteins, HERC3, KCNN2, MRPL52 and NOMO2 (Additional file 6: Figure S6C). HERC3, MRPL52 and NOMO2 were decreased in both PCa samples and LNCaP cells under antiandrogen treatment, while KCNN2 was increased in PCa samples and reduced in LNCaP cells (Fig. 6C, D). Interestingly, expression levels of HERC3, KCNN2, and MRPL52 were not significantly varied in tumor group and control group (Additional file 6: Figure S6D). In addition, the mRNA expression levels of *HERC3, KCNN2, MRPL52* and *NOMO2* were all boosted in primary PCa according to TCGA data (Fig. 6E). Also, the binding of AR to transcriptional start site (TSS) of *HERC3* and promoter of *KCNN2* indicated the transcriptional regulation of AR to HERC3 and KCNN2 (Fig. 6F, Additional file 6: Figure S6E). In addition, the protein expression of HERC3 was decreased in LNCaP cells with bicalutamide treatment and was no change in PC3 cells (Fig. 7). However, there is no AR binding in the vicinity of promoter regions of *MRPL52* and *NOMO2*, and diminished protein levels under enzalutamide treatment implied complicated regulation of two proteins (Fig. 6D, Additional file 6: Figure S6F, S6G). Collectively, these four proteins have the potential to be biomarkers adjusted by antiandrogens.

## Discussion

Proteins execute vital functions in living creatures, and protein homeostasis is crucial to maintaining health of human body. Therefore, inspecting the changes in the proteome in PCa could not only discover the biomarkers of tumor but also help to explore molecular mechanisms of tumorigenesis and progression. Also, sample preservation and quantitative mass spectrometric methods are important to the depth of proteomics. Recently, a study performed the proteomics of PCa to identify several proteins associated with patients with biochemical recurrence [25]. By using formalin-fixed tumor tissue and DIA-NN, 5,803 proteins were quantified in the study. In our cohort, we used snap-frozen PCa samples from China to perform DIA-PASEF proteomics, identifying 8,771 proteins, which could provide the most abundant and integral information about proteome [28]. Two proteins, CKM and COMP, were revealed to have the potential to be diagnostic biomarkers at both mRNA and protein levels (Additional file 2: Figure S2B, S2K). CKM, an M-type creatine kinase, is highly expressed in heart, esophagus, and prostate [29]. CKM catalyzes the transfer of the phosphoryl group between ATP and phosphocreatine, contributing to energy consumption for heart [30]. A decrease in CKM activity due to acetylation may exacerbate high-energy phosphoryl transfer in the context of heart failure [31]. So far, there has no report about role of CKM in prostate. We first reported down-regulation of CKM in PCa, and based on its function in heart, we extrapolated that decreased CKM could weaken energy transfer and the function of prostate muscle. COMP, an ECM protein, was reported as a prognostic factor in colorectal cancer [32], favored the development and metastasis of breast cancer [33], and drove hepatocellular carcinoma progression [34]. Here we present that COMP was increased and might be a diagnostic biomarker of PCa, while the exact action of COMP remained unknown in PCa.

Proteostasis in normal cells maintains healthy development and senescence, whereas dysfunction of proteostasis in cells could cause many disorders, including tumors [35]. E3 ubiquitin ligase and DUBs participate in the regulation of proteostasis in cells [36–39]. Therefore, it would be worthwhile to explore the changes of E3 ligase and DUBs in PCa. In this study, we detected several down-regulated E3 ligases and a DUB in our cohort (Fig. 2A and G) and uncovered that DUB UCHL1 and E3 ligase PJA2 have the potential to be diagnostic biomarkers in mRNA level (Additional file 3: Figure S3A, S3F). UCHL1 demonstrated dual roles in different tumors: as an oncogene in lymphoma [40], colorectal cancer [41] and B-cell [42], as a tumor suppressor in nasopharyngeal carcinoma [43], PCa [44], and ovarian cancer [45]. We noticed UCHL1 was diminished at both mRNA and protein levels in PCa. However, the ROC analysis of UCHL1 protein did not present statistical significance (data not shown), which should be further explored for IHC staining in a large cohort. PJA2 could promote ubiquitination of KSR1 [46], MOB1 [47], and MFHAS1 [48]. The role of PJA2 in PCa is not known yet. Overall, there are no known interacting partners for E3 ligases PJA2, ZNF451, FBXO2, DCAF10, and DUB UCHL1 in our csDEPs due to the limitation of samples and technology (Fig. 1F), but this phenomenon does not mean that down-regulated UCHL1, PJA2, ZNF451, FBXO2, and DCAF10 have no effects on proteostasis in PCa.

The concentration of antiandrogen drugs used in vitro and in vivo is important to their efficacy. Enzalutamide dose (10µM) is based on its IC50 to cells (Additional file 4: Figure S4A). The drug dose in clinical practice is according to National Comprehensive Cancer Network (NCCN) guidelines (50 mg/day for Bicalutamide, 160 mg/day for Enzalutamide). The higher concentration of enzalutamide than bicalutamide in clinical usage indicates the complexity of the drug’s dosing. According to the literature [49], plasma concentrations for bicalutamide could be 9 µg/mL (20.9µM), which is twice the concentration of our usage in vitro and a higher plasma concentration could increase the bioavailability of the drug.

The incidence of PCa rises with advancing age. However, the role of age as a prognostic factor for biochemical recurrence (BCR) after radical prostatectomy remains controversial [50–53]. In our small cohort, we analyzed the effect of age on biochemical recurrence and found that there was no statistically significant difference observed in relation to biochemical recurrence (*P* = 0.6395) or Gleason grades (GGs) (*P* = 0.8988) (Additional file 7: Table S9). Additionally, comparing the BCR group and the no-recurrence group, no significance was noticed in the age group (*P* = 0.5008) or GGs (*P* = 0.0826). Interestingly, based on Gleason score, the BCR group had a significantly higher score (*P* = 0.0325) (Additional file 7: Table S10). This result indicated that age is not an independent prognostic factor in our cohort. Further investigation is required to determine the conclusion and potential impact of age as a confounding variable, utilizing a sizable cohort study with an extended duration of follow-up.

Deep detection of proteome alteration caused by antiandrogen treatment is an important approach to scrutinizing the molecular mechanism of drug resistance in PCa. Up-regulated H2AX was identified in both LNCaP cells and PC3 cells under enzalutamide treatment (Fig. 4F and Additional file 5: Figure S5F), which is not detected in clinical samples, indicating an AR-independent effect of DNA damage by antiandrogens. DUB OTUD5 was found to be decreased in both LNCaP cells and PC3 cells (Fig. 5A and C), not in clinical samples. As UCHL1, OTUD5 demonstrated oncogenic roles in colon cancer and breast cancer, and suppressing features in HCC and cervical cancer [54]. The function of OTUD5 in PCa needs to be further investigated.

When lnDEPs and DEPs from patients receiving antiandrogen intervention were compared, HERC3, KCNN2, MRPL52, and NOMO2 levels in both DEPs were significantly altered. ChIP-seq data of AR manifested AR binds to the promoter regions of HERC3 and KCNN2. These data suggest that the effect of antiandrogens on PCa cells could be both AR-dependent and AR-independent. Therefore, comprehensive perspectives should be taken to understand the drug resistance to antiandrogens in PCa.

## Conclusions

Our study has identified two potential diagnostic makers of PCa and four significantly changed proteins in PCa upon antiandrogen treatment using DIA-PASEF proteomics. These indicative findings may benefit the diagnosis of PCa and provide insights into the development of novel therapeutic drugs to improve antiandrogen intervention.

### Electronic supplementary material

Below is the link to the electronic supplementary material.

Supplementary Material 1

Supplementary Material 2

Supplementary Material 3

Supplementary Material 4

Supplementary Material 5

Supplementary Material 6

Supplementary Material 7

Supplementary Material 8

Supplementary Material 9

Supplementary Material 10

Supplementary Material 11

Supplementary Material 12

Supplementary Material 13

Supplementary Material 14

Supplementary Material 15

Supplementary Material 16

Supplementary Material 17

## Data Availability

All proteomics data in this study have been deposited to the ProteomeXchange Consortium (https://proteomecentral.proteomexchange.org) via the iProX partner repository with the dataset identifier PXD046769.

## Abbreviations

PCa: Prostate cancer
AR: Androgen receptor
DIA: Data-independent acquisition
PASEF: Parallel accumulation serial fragmentation
CKM: M-type creatine kinase
COMP: Cartilage oligomeric matrix protein
ECM: Extracellular matrix
DUB: Deubiquitinating enzyme
PSA: Prostate-specific antigen
E3 ligome All: E3 ubiquitin ligases in cell
DUBome: All deubiquitinating enzymes in cell
CRPC: Castration-resistant prostate cancer
BCA: Bicinchoninic acid
TEAB: Tetraethylammonium bromide
DTT: Dithiothreitol
IAA: Iodoacetamide
LC-MS/MS: Liquid chromatography-mass spectrometry/mass spectrometry
HCD: Higher energy collisional dissociation
NCE: Normalized collision energy
AGC: Automatic gain control
FC: Fold change
DEPs: Differential expression proteins
GO: Gene ontology
KEGG: The Kyoto Encyclopedia of Genes and Genomes
PPI: Protein-protein interactions
LFQ: Label-free Quantification
TCGA: Cancer Genome Atlas
csDEPs: Clinical sample DEPs
MCL: Markov cluster algorithm
UMAP: Uniform manifold approximation and projection
lnDEPs: DEPs in LNCaP cells
pDEPs: DEPs in PC3 cells
