# Supplementary figures and images for: Proteomic profiling of prostate cancer reveals molecular signatures under antiandrogen treatment
Source: Clin Proteomics. 2024 Jun 26;21:44. doi: 10.1186/s12014-024-09490-9 (PMC11202386; doi:10.1186/s12014-024-09490-9)

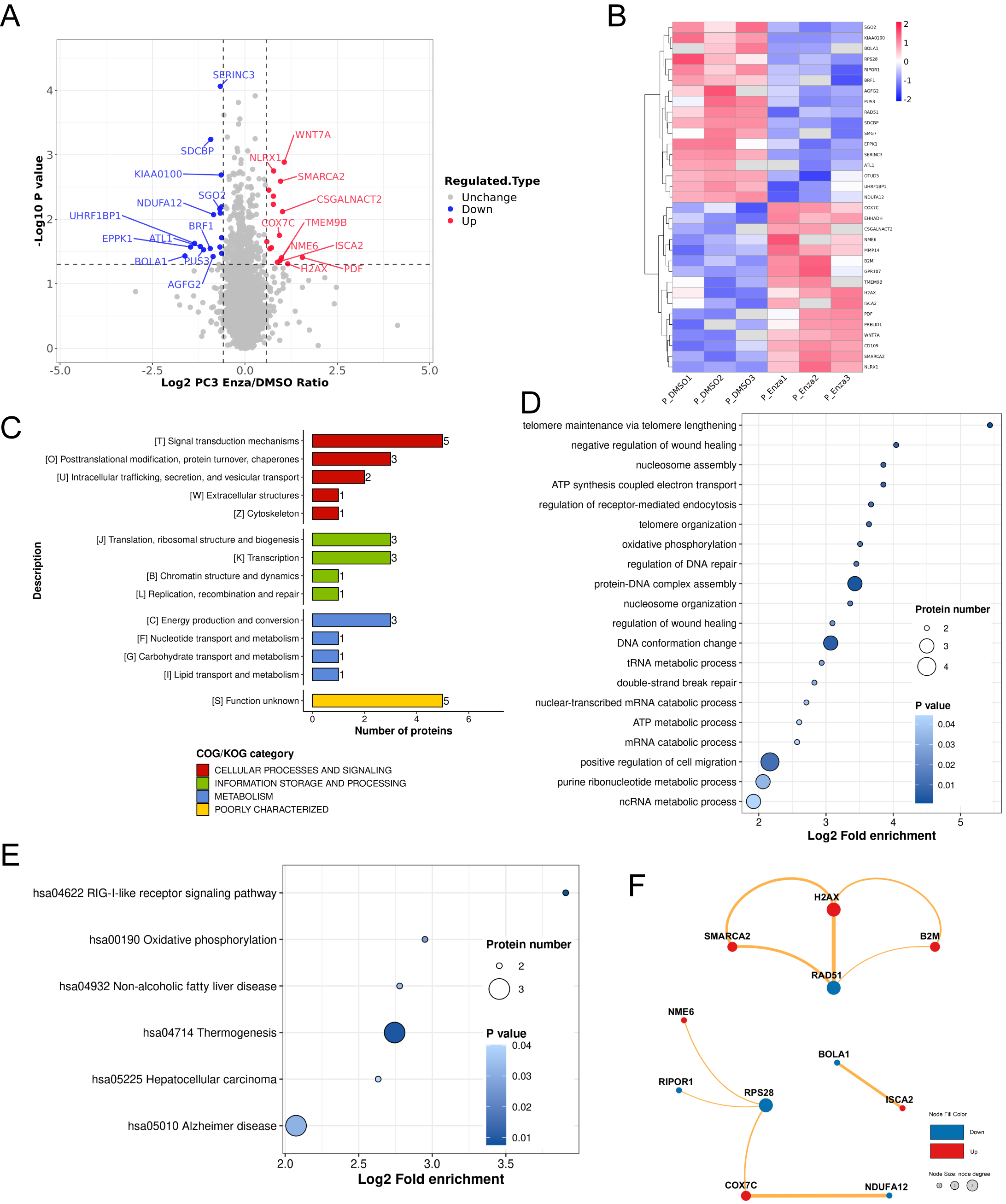

Supplement: Supplementary file 1 — Supplementary Material 1 [file 12014_2024_9490_MOESM1_ESM.png]

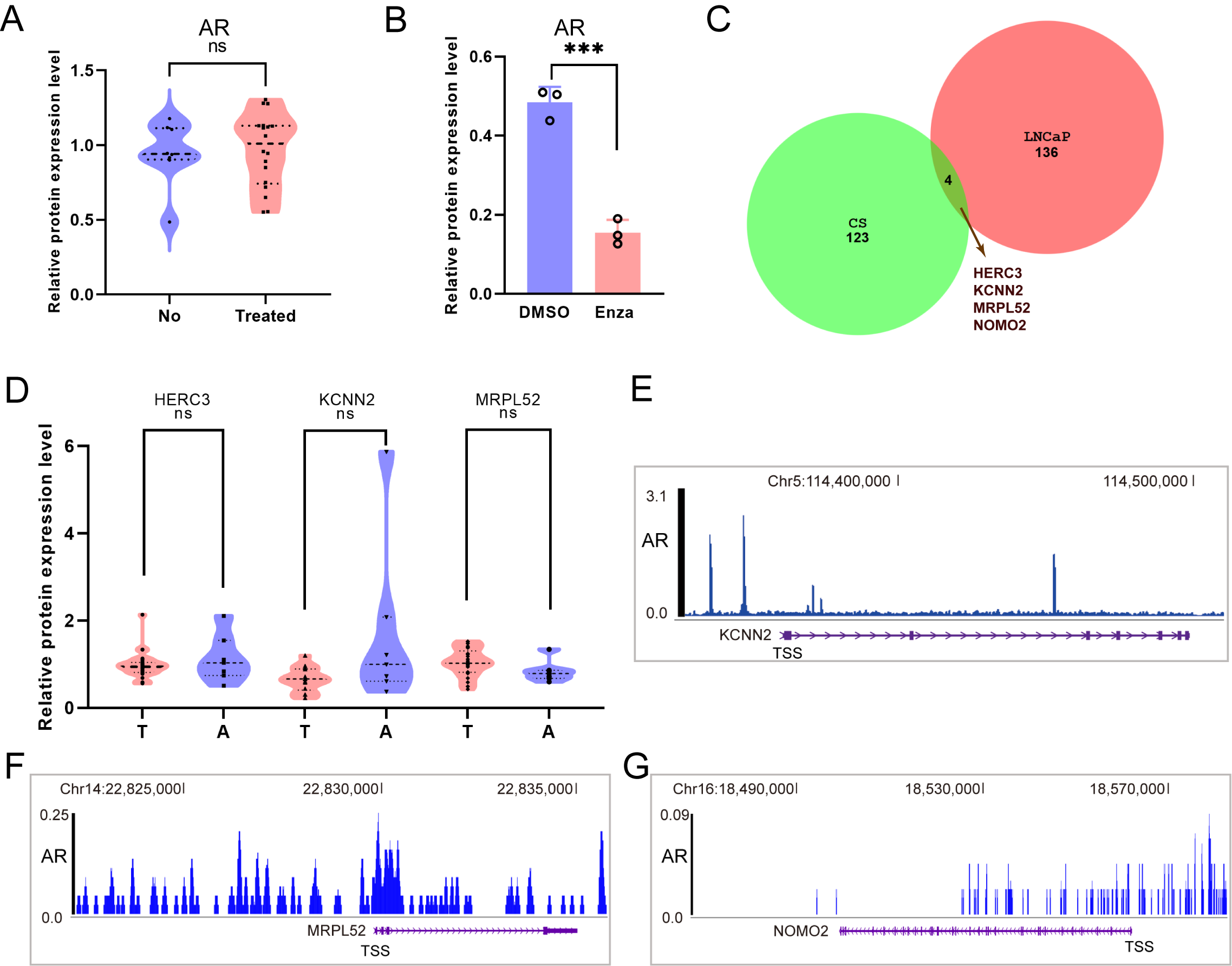

Supplement: Supplementary file 2 — Supplementary Material 2 [file 12014_2024_9490_MOESM2_ESM.png]

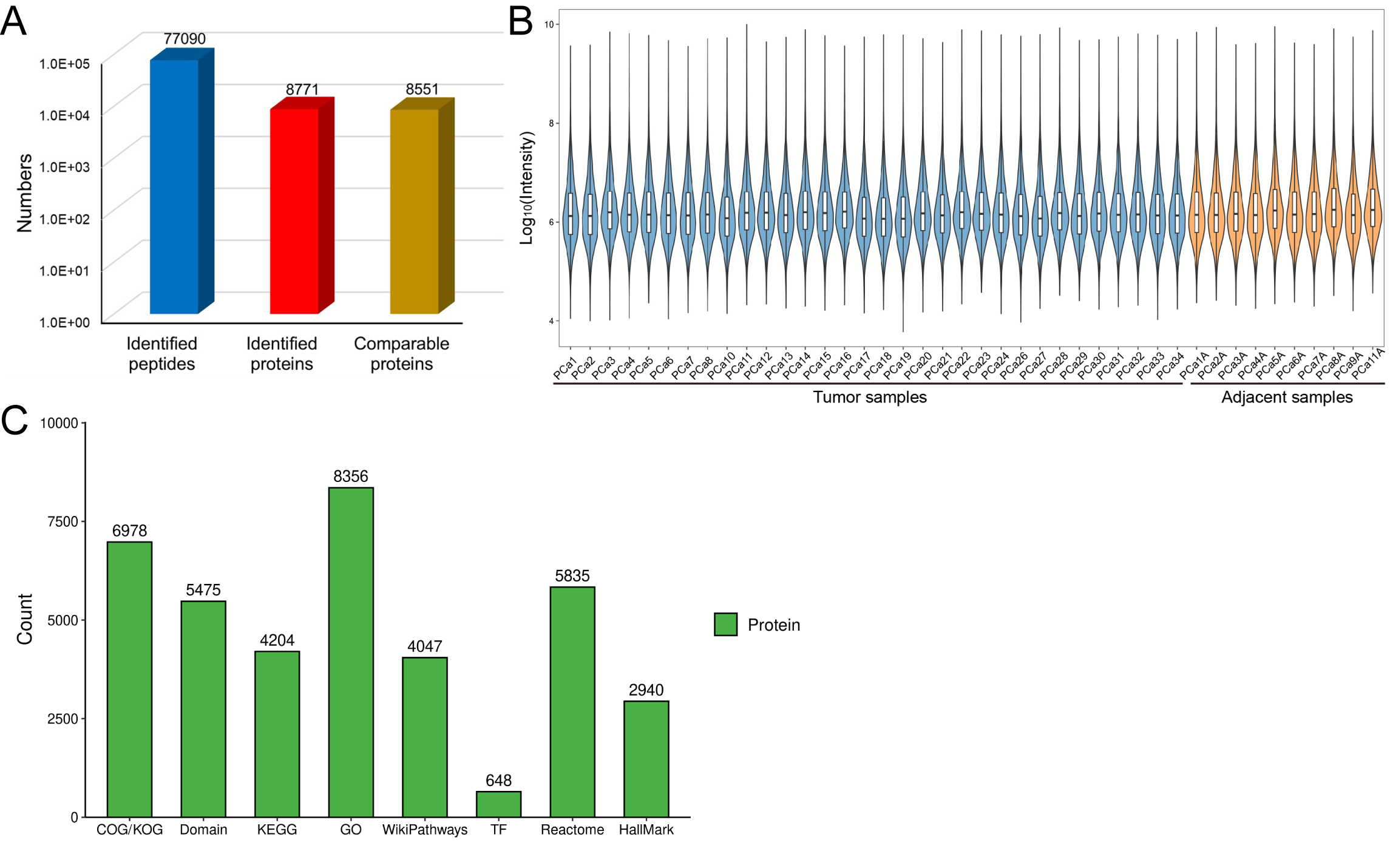

Supplement: Supplementary file 10 — Supplementary Material 10 [file 12014_2024_9490_MOESM10_ESM.png]

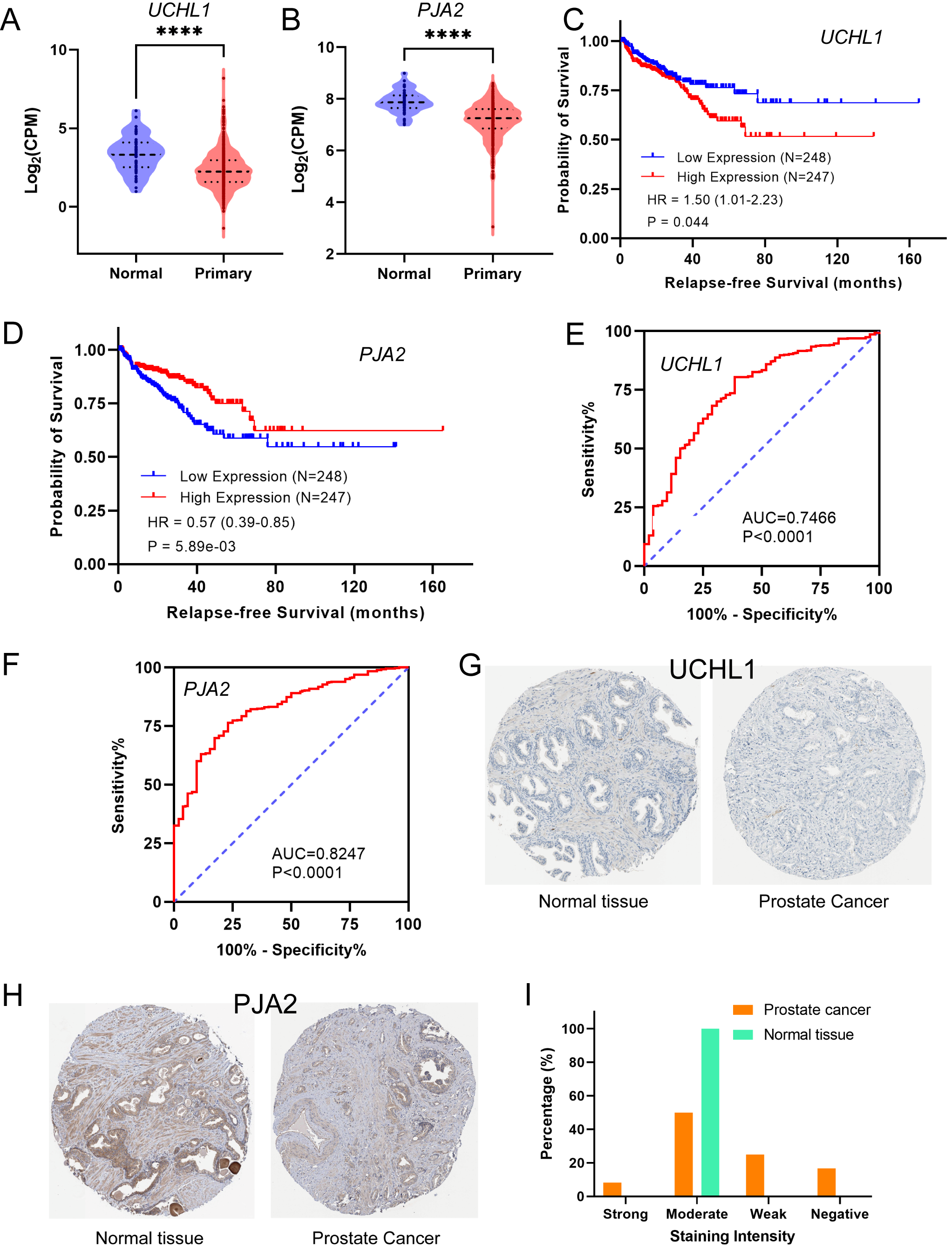

Supplement: Supplementary file 11 — Supplementary Material 11 [file 12014_2024_9490_MOESM11_ESM.png]

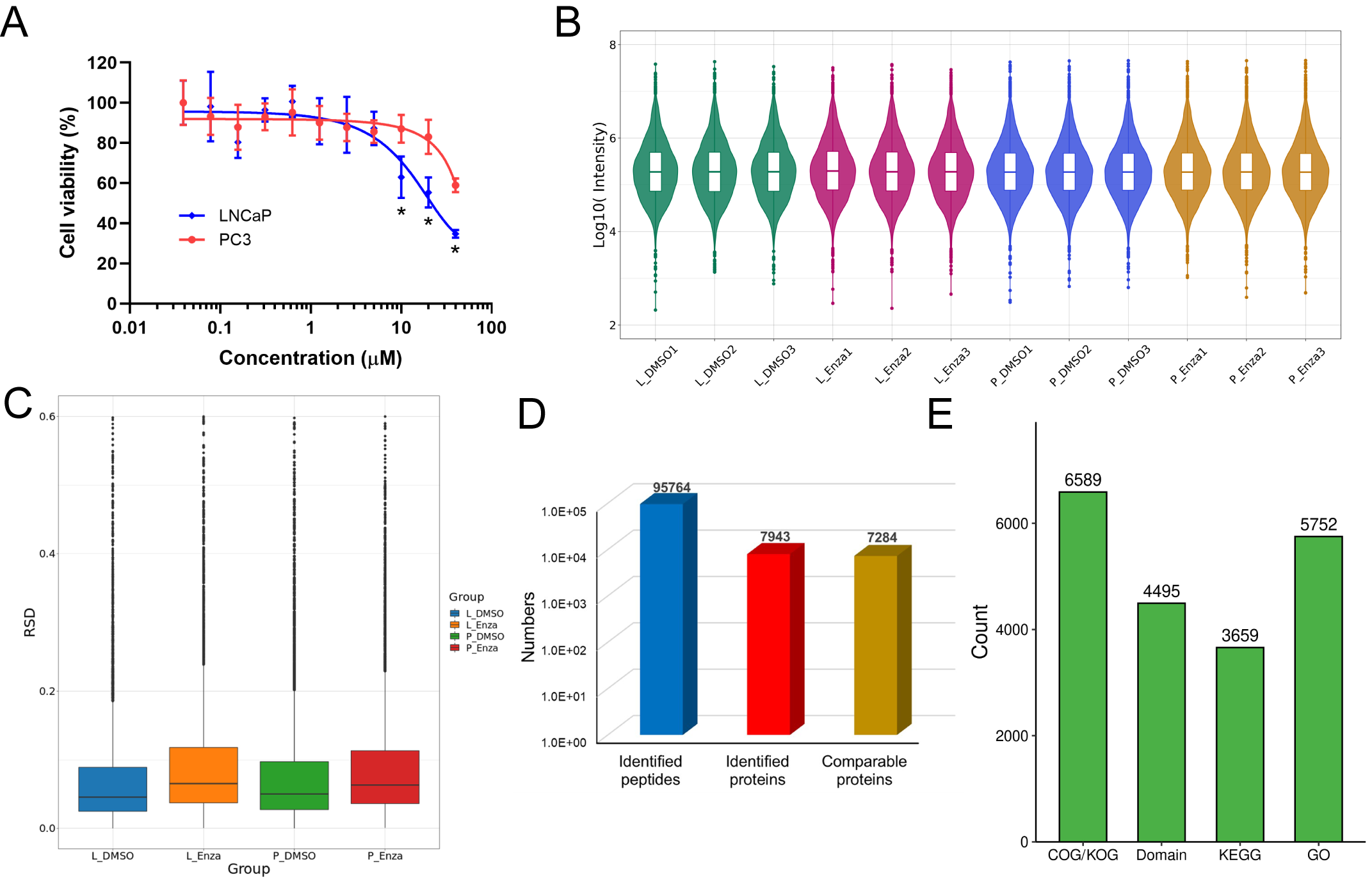

Supplement: Supplementary file 12 — Supplementary Material 12 [file 12014_2024_9490_MOESM12_ESM.png]

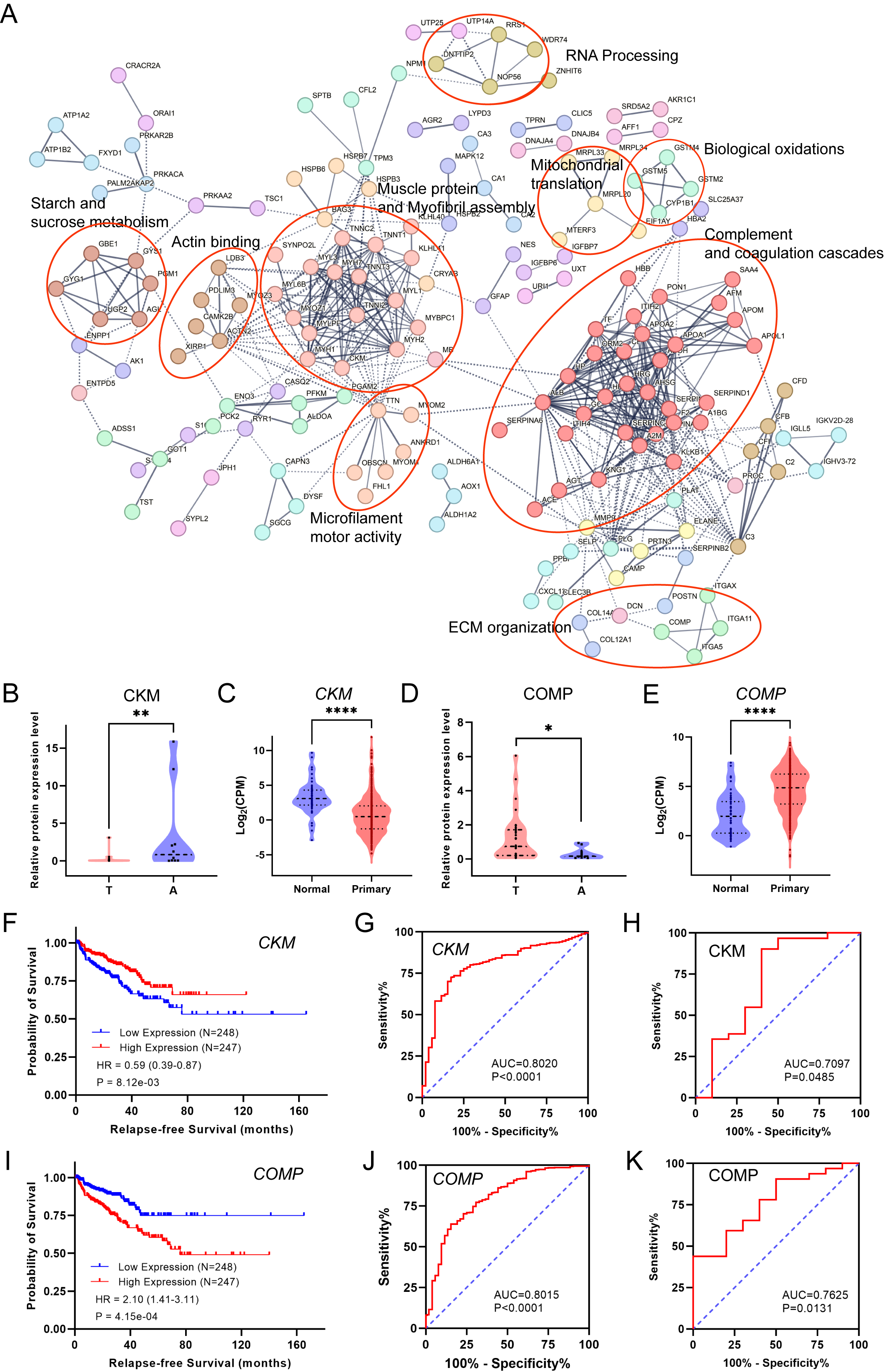

Supplement: Supplementary file 13 — Supplementary Material 13 [file 12014_2024_9490_MOESM13_ESM.png]
