# Supplementary material for: Proteomic profiling of prostate cancer reveals molecular signatures under antiandrogen treatment
Source: Clin Proteomics. 2024 Jun 26;21:44. doi: 10.1186/s12014-024-09490-9 (PMC11202386; doi:10.1186/s12014-024-09490-9)
Supplement: Supplementary file 14 — Supplementary Material 14 [file 12014_2024_9490_MOESM14_ESM.docx]

Table S1. Clinicopathological features of patients with prostate cancer (PCa).

| No. of patients | 32 (32 tumors and 10 adjacents) | | |  |
| --- | --- | --- | --- | --- |
| No. of patients with follow-up | 25 | | |  |
| Median follow-up (months) | 15.5 | | | |
| Range (months) | 0-19 | | | |
| Clinicopathological characteristics | | | |  |
| Variable | | **n** | **%** |  |
| Age at diagnosis (median = 68.5 years, range = 44-84) | | | |  |
| <68 | | 16 | 50 |  |
| ≥68 | | 16 | 50 |  |
| Gleason grade | | | |  |
| GG1 | | 3 | 9.4 |  |
| GG2 | | 4 | 12.5 |  |
| GG3 | | 3 | 9.4 |  |
| GG4 | | 5 | 15.6 |  |
| GG5 | | 17 | 53.1 |  |
| Tumor stage (TNM) | | | |  |
| I | | 3 | 9.4 |  |
| II | | 8 | 25.0 |  |
| III | | 3 | 9.4 |  |
| IV | | 18 | 56.2 |  |
| Surgical margin | | | |  |
| Negative | | 13 | 40.6 |  |
| Positive | | 6 | 18.8 |  |
| Unknown | | 13 | 40.6 |  |
| Tumor percentage | | | |  |
| 50% | | 1 | 3.1 |  |
| 60% | | 4 | 12.5 |  |
| 70% | | 14 | 43.8 |  |
| 80% | | 8 | 25.0 |  |
| 90% | | 5 | 15.6 |  |
| Biochemical recurrence events | | | |  |
| Missing | | 7 | 21.9 |  |
| No | | 20 | 62.5 |  |
| Yes | | 5 | 15.6 |  |
| Type of sampling | | | |  |
| Radical prostatectomy | | 19 | 59.4 |  |
| Prostate biopsy | | 13 | 40.6 |  |
| Bicalutamide treatment | | | |  |
| Yes | | 22 | 68.8 |  |
| No | | 10 | 31.2 |  |
| Bicalutamide treatment response (out of 22) | |  |  |  |
| PSA decrease | | 14 | 63.6 |  |
| PSA no response | | 0 | 0 |  |
| No detection | | 8 | 36.4 |  |
