# Supplementary material for: Proteomic profiling of prostate cancer reveals molecular signatures under antiandrogen treatment
Source: Clin Proteomics. 2024 Jun 26;21:44. doi: 10.1186/s12014-024-09490-9 (PMC11202386; doi:10.1186/s12014-024-09490-9)
Supplement: Supplementary file 15 — Supplementary Material 15 [file 12014_2024_9490_MOESM15_ESM.docx]

Table S9. Comparison between age groups.

|  | **<68 years old**  **(n=16)** | **>68 years old**  **(n=16)** | ***P* Value** |
| --- | --- | --- | --- |
| **Biochemical recurrence (BCR), n(%)** | 3 (18.75) | 2 (12.5) | 0.6395 |
| **Gleason grade (GGs), n(%)** |  |  | 0.8988 |
| **1** | 2 (12.5) | 1 (6.25) |  |
| **2** | 1 (6.25) | 1 (6.25) |  |
| **3** | 1 (6.25) | 4 (25) |  |
| **4** | 3 (18.75) | 2 (12.5) |  |
| **5** | 9 (56.25) | 8 (50) |  |
| **Gleason score, n(%)** |  |  | 0.8903 |
| **6** | 2 (12.5) | 1 (6.25) |  |
| **7** | 2 (12.5) | 5 (31.25) |  |
| **8** | 3 (18.75) | 2 (12.5) |  |
| **9** | 8 (50) | 4 (25) |  |
| **10** | 1 (6.25) | 4 (25) |  |
