# Supplementary material for: Proteomic profiling of prostate cancer reveals molecular signatures under antiandrogen treatment
Source: Clin Proteomics. 2024 Jun 26;21:44. doi: 10.1186/s12014-024-09490-9 (PMC11202386; doi:10.1186/s12014-024-09490-9)
Supplement: Supplementary file 16 — Supplementary Material 16 [file 12014_2024_9490_MOESM16_ESM.docx]

Table S10. Comparison between BCR and no BCR groups.

|  | **Biochemical recurrence (BCR)**  **(n=5)** | **No recurrence**  **(n=20)** | ***P* Value** |
| --- | --- | --- | --- |
| **Age ± SD** | 65 ± 12.3 | 68 ± 7.8 | 0.5008 |
| **GGs, n(%)** |  |  | 0.0826 |
| **1** | 0 | 2 (10) |  |
| **2** | 0 | 1 (5) |  |
| **3** | 0 | 3 (15) |  |
| **4** | 0 | 5 (25) |  |
| **5** | 5 (100) | 9 (45) |  |
| **Gleason score, n(%)** |  |  | 0.0325 |
| **6** | 0 | 2 (10) |  |
| **7** | 0 | 4 (20) |  |
| **8** | 0 | 5 (25) |  |
| **9** | 3 (60) | 7 (35) |  |
| **10** | 2 (40) | 2 (10) |  |
