# Supplementary material for: Proteomic profiling of prostate cancer reveals molecular signatures under antiandrogen treatment
Source: Clin Proteomics. 2024 Jun 26;21:44. doi: 10.1186/s12014-024-09490-9 (PMC11202386; doi:10.1186/s12014-024-09490-9)
Supplement: Supplementary file 17 — Supplementary Material 17 [file 12014_2024_9490_MOESM17_ESM.docx]

**Supplemental Figure Legends**

**Fig. S1** Overview of the proteomic profile of PCa. **A** Number of identified peptides and proteins in PCa samples. **B** Distribution of protein intensity of all PCa samples. **C** Protein numbers of the functional annotation of identified proteins of PCa in different databases.

**Fig. S2** CKM and COMP were potential diagnostic biomarkers at both mRNA and protein levels for PCa. **A** PPI network of csDEPs, clustered by STRING using MCL clustering method. Main clusters were circled and labeled. The dotted line showed the edges between clusters. **B** Expression levels of CKM protein in our PCa samples and adjacent tissues. **C** Expression levels of *CKM* mRNA in normal prostate tissue and primary PCa in TCGA PRAD cohort. **D** Expression levels of COMP protein in our PCa samples and adjacent tissues. **E** Expression levels of *COMP* mRNA in normal prostate tissue and primary PCa in TCGA PRAD cohort. **F** and **I** Kaplan-Meier relapse-free survival (RFS) analyses of patients with PCa based on *CKM* and *COMP* expression in TCGA PRAD database. **G** and **J** Receiver operating characteristic (ROC) analyses demonstrated the diagnostic achievements of *CKM* and *COMP* mRNAs in TCGA PRAD database. **H** and **K** ROC analyses presented diagnostic performances of CKM and COMP proteins in our PCa samples.

**Fig. S3** The mRNAs of UCHL1 and PJA2 have the potential to be biomarkers for PCa. **A** and **B** mRNA expression levels of *UCHL1* and *PJA2* in normal prostate tissue and primary PCa in TCGA PRAD cohort. **C** and **D** Kaplan-Meier relapse-free survival (RFS) analyses of patients with PCa according to *UCHL1* and *PJA2* expression in TCGA PRAD database. **E** and **F** Receiver operating characteristic (ROC) analyses demonstrated the diagnostic achievements of *CKM* and *COMP* mRNAs in TCGA PRAD database. **G** Representative images of IHC staining of UCHL1 in normal prostate tissue and PCa from HPA. Image credit: human protein atlas. **H** Representative images of IHC staining of PJA2 in normal prostate tissue and PCa from HPA. Image credit: human protein atlas. **I** IHC staining intensity for images in H.

**Fig. S4** Outline of the proteome profile of PCa cells with enzalutamide treatment. **A** IC50 of enzalutamide in LNCaP cells and PC3 cells. **B** Distribution of protein intensity of every cell sample. **C** Relative standard deviation (RSD) analysis of replicates of cell samples. **D** Number of identified peptides and proteins in PCa cells. **E** Protein numbers of the functional annotation of identified proteins of PCa cells in different databases.

**Fig. S5** Proteomic details of PC3 cells treated with enzalutamide. **A** Volcano plot of all identified proteins in PC3 cells. Red dots for up-regulated proteins and blue dots for down-regulated proteins. The top changed proteins were labeled. **B** Heatmap of pDEPs. **C** COG/KOG classification of pDEPs. **D** Dot plot of gene ontology (GO) enrichment of pDEPs in biological process (BP). **E** Dot plot of pathway enrichment of pDEPs in KEGG pathway. **F** Protein-protein interaction (PPI) network of pDEPs created by Cytoscape. pDEPs, differential expression proteins in PC3 cells.

**Fig. S6** Several characteristics of potential prediction biomarkers for antiandrogen-treated PCa. **A** Protein expression level of AR in PCa samples with bicalutamide treatment. **B** Expression level of AR protein in LNCaP cells. **C** Venn diagram of DEPs in clinical samples and LNCaP cells found four proteins, HERC3, KCNN2, MRPL52, and NOMO2. **D** Expression levels of HERC3, KCNN2, and MRPL52 proteins in PCa. **E-G** AR occupancy at the putative promoter regions of *KCNN2*, *MRPL52*, and *HERC3* in the WashU browser for AR ChIP-seq data.
